# Updated Mortality Analysis of SELTINE, the French Cohort of Nuclear Workers, 1968–2014

**DOI:** 10.3390/cancers15010079

**Published:** 2022-12-23

**Authors:** Olivier Laurent, Eric Samson, Sylvaine Caër-Lorho, Lucie Fournier, Dominique Laurier, Klervi Leuraud

**Affiliations:** Ionizing Radiation Epidemiology Laboratory (LEPID), Institute for Radiobiological Protection and Nuclear Safety (IRSN), 92262 Fontenay-aux-Roses, France

**Keywords:** epidemiology, ionizing radiation, low dose, nuclear workers

## Abstract

**Simple Summary:**

Cohort studies of nuclear workers are key to study the health effects of protracted exposures to low doses of ionizing radiation (IR), which is necessary to verify the adequacy of radiation protection standards. In this cohort of nuclear workers monitored for exposure to IR and followed-up for mortality, a strong healthy worker effect was observed. Exposure to IR was significantly associated with death from leukaemia and dementia. This second finding is novel and should be interpreted with caution at this stage. It should motivate the research community to evaluate its replicability by studying dementia in other cohorts exposed to low-dose IR. Findings for solid cancers were not statistically significant, but estimates were still imprecise and compatible with those from other studies. Continued follow-up of the cohort and its participation in pooled analyses with similar cohorts will help to further improve the statistical precision of risk estimates in the future.

**Abstract:**

Cohorts of nuclear workers are particularly relevant to study the health effects of protracted exposures to low doses at low dose-rates of ionizing radiation (IR). In France, a cohort of nuclear workers badge-monitored for external IR exposure has been followed-up for several decades. Its size and follow-up period have recently been extended. The present paper focuses on mortality from both cancer and non-cancer diseases in this cohort. The SELTINE cohort of nuclear workers employed by CEA, Orano, and EDF companies was followed-up for mortality from 1968 to 2014. Mortality in the cohort was compared to that in the French general population. Poisson regression methods were used to estimate excess relative rates of mortality per unit of cumulative dose of IR, adjusted for calendar year, age, company, duration of employment, and socioeconomic status. The cohort included 80,348 workers. At the end of the follow-up, the mean attained age was 63 years, and 15,695 deaths were observed. A strong healthy worker effect was observed overall. A significant excess of pleural cancer mortality was observed but not associated with IR dose. Death from solid cancers was positively but non-significantly associated with radiation. Death from leukaemia (excluding chronic lymphocytic leukaemia), dementia, and Alzheimer’s disease were positively and significantly associated with IR dose. Estimated dose–risk relationships were consistent with those from other nuclear worker studies for all solid cancers and leukaemia but remained associated with large uncertainty. The association between IR dose and dementia mortality risk should be interpreted with caution and requires further investigation by other studies.

## 1. Introduction

Ionizing radiation (IR) is an established carcinogenic agent [1]. The effects of IR on cancer risks are well established for dose levels above 100 milliGrays (mGy) [2]. This is well documented, especially for external exposure to photons, thanks to the epidemiological follow-up of Hiroshima and Nagasaki atomic bomb survivors (A-Bomb survivor study) who experienced quasi-instantaneous exposure to IR [3]. A growing number of other epidemiological studies covering various exposure situations also contributed to the knowledge on the relationships between IR exposure and incidence of various cancers [4,5]. However, questions remain about the shape of dose–risk relationships for cancers below 100 mGy, corresponding to the so-called low-dose domain [2]. 

Existence of radiation-related risks of circulatory diseases is well established for exposures to IR at doses above 500 mGy [2,6]. Some studies reported associations for lower levels of exposure [7,8], but the results remain highly heterogeneous and call for confirmation from other studies [9]. Open questions also exist on possible effects of low doses of IR on other non-cancer diseases, e.g., metabolic [6] or neurocognitive [10,11] diseases.

Epidemiological cohort studies of nuclear workers are key to provide knowledge on the potential health effects of low dose, protracted exposure to IR because these workers have generally accumulated low doses during their career and since these doses have been monitored by occupational medicine for decades [12,13,14]. These cohorts allow for study of both cancer [15] and non-cancer diseases [7], generally as causes of death but also sometimes as incidence data [16,17,18]. As all the cohorts were designed to study long-term effects of exposures, these studies are increasingly informative as time goes by and their follow-up extends. Such studies have been conducted in most countries hosting major nuclear industries [12], including in France.

The French nuclear program was developed at the end of World War II and was more widely deployed from the 1950s. Forty years later, two epidemiological studies were set up to evaluate health effects of protracted exposure to low-dose external IR in nuclear workers employed by the French Atomic Energy Commission (CEA; today, French Alternative Energies and Atomic Energy Commission) or the Cogema company (Orano today) [19,20,21] on the one hand and by the Electricité de France (EDF) company [22,23] on the other hand. CEA is a large research organisation in the fields of defence and security, low-carbon energies, physical science, and life sciences; Orano conducts activities related to the uranium fuel cycle, i.e., mining, fuel production and enrichment, processing and recycling of spent fuel, and clean-up and dismantling, and EDF produces and supplies electricity, with 19 nuclear power plants in 2022. Data from both studies were integrated into the collaborative 15-country study that reported a dose-related increase in all cancer mortality [12], at that time with a follow-up period of 1968–1994 for both French cohorts. 

Since then, a French national cohort of more than 59,000 workers has been constituted by merging the two initial cohorts. This cohort was analysed by the Institute for Radiological Protection and Nuclear Safety (IRSN) [24], with a follow-up period of 1968–2004, yielding information on cancer mortality risk that was consistent with conclusions from other nuclear studies published during the same period [16,25]. These studies are based on a common criterion, namely that these workers had to be regularly badge-monitored as part of radiation protection monitoring. The French nuclear worker cohort participated in the international INWORKS collaboration, which estimated dose-related risk coefficients with improved precision compared to the 15-country study because of a longer follow-up and a higher number of observed deaths from cancer [13,15,26] and non-cancerous diseases [7]. 

The French cohort of nuclear workers is now denominated SELTINE (for Suivi Epidémiologique Longitudinal des Travailleurs de l’Industrie Nucléaire française). Its size has been recently enlarged by integration of additional workers, and its follow-up has been further extended. In the present analysis, the mortality of French nuclear workers is examined over the 1968–2014 period, and associations between protracted exposure to low-dose IR and mortality from both cancer and non-cancer diseases are investigated.

## 2. Materials and Methods

### 2.1. Study Population

SELTINE assembles data on workers hired: 1) between January 1, 1950 and December 31, 2004 by CEA, between January 1, 1976 and December 31, 2004 by Orano (formerly Cogema 1976–2005, then AREVA NC 2006–2017), or between January 1, 1961 and December 31, 2003 by EDF, 2) who have or had been employed (permanent contract) for at least 365 days, and 3) who were badge-monitored in the course of their career at these companies. Another inclusion criterion was to be alive on January 1, 1968 because individual medical causes of death are only available from this date in France (see below). Workers known to have been involved in uranium mining or milling activities, identified either from administrative files or dosimetry recordings, were excluded from SELTINE because their exposure is mainly due to radon gas and its progenies [27] or has not been completely recorded [28]. These workers are included in other epidemiological studies [27,28]. Compared to the previous study, not only was the hiring period extended but CEA workers monitored in nuclear research sites for military applications before 1995 (about 12,000 workers) were also included because reconstruction of their dosimetry records has been completed. 

Administrative data needed to identify eligible workers and to reconstruct their occupational histories, i.e., employment periods, workplace locations, were provided by the CEA, Orano, and EDF. It also informed on job title at hiring in the company, which was used to assign a socioeconomic status indicator to each worker: manager or engineer/administrative employee/skilled worker/unskilled worker. If dosimetry records were identified outside the employment periods at the three companies, the date of hire or date of termination was changed accordingly and the worker was considered employed by a subsidiary. As workers may have had multiple employers during the follow-up period, a fixed variable was created to identify the company for which the worker had worked the longest: CEA, Orano, EDF, or other for subsidiaries. In the event of a tie, the company with the oldest period of employment was retained. Duration of employment was defined as a time-dependent variable that increased from the date of hiring until either the date of termination of employment (and remained at that level thereafter) or the end of follow-up if it occurred during active employment.

### 2.2. Vital Status and Outcome Determination

Vital statuses were ascertained by linking the cohort with the French National Directory for the Identification of Natural Persons (RNIPP), which is maintained by the National Institute of Statistics and Economic Studies (Insee) and gathers information on the vital status of French citizens. Workers who could not be linked to the RNIPP were considered lost to follow-up. Individual causes of death were obtained from the French national registry maintained by the Epidemiology Centre on the medical Causes of Death (CépiDC) of the French National Institute of Health and Medical Research (Inserm). The CépiDC has been registering individual medical causes of death in France since January 1, 1968. Causes of death were coded according to the International Classification of Disease (ICD) in effect at the time of death: ICD8 from 1968 through 1978, ICD9 from 1979 through 1999, and ICD10 from 2000 on (Supplementary Table S1). The mortality follow-up began at the latest of the three following dates: 1 year after the date of first hire, the date of the first dosimetry monitoring, or January 1, 1968. The follow-up ended at the date of death or December 31, 2014, whichever occurred first. For workers lost to follow-up, it ended at the date of the most recent news, based either on the administrative data provided by the companies, on the date of the last dosimetry record, or on the result of the previous vital status search.

### 2.3. Radiation Dose Reconstruction

Dose equivalents of external radiation (primarily photons) recorded by personal dosimeters worn on the chest were provided for each worker and each year from 1950 to 2004 (2003 for EDF) by companies’ dosimetry laboratories [19,23,24], then by linkage with SISERI, the French information system for monitoring exposure of radiation workers [29], from 2005 (2004 for EDF) to 2014. The nature of the reported doses has evolved over time: equivalents in soft tissue at a depth of 3 mm (Dt(3)), then at a depth of 10 mm (Hp(10)), as well as the units in which these doses were expressed (rem, Sievert (Sv)), requiring homogenization over time and sites.

As part of two previous international studies [30,31], data were collected for the period 1950–2004 on the type of dosimeter used, exposure conditions, and dosimeter response. Dose correction coefficients were calculated over the different periods and for the different nuclear facilities to correct reported doses for the influence of dosimeter wearing conditions (responses to different energies and dose geometries) and the effect of the radiation spectrum at the workstation [31]. After 2004, changes in dosimeters occurred at CEA and EDF: at CEA, the IRSN radio photo luminescent (RPL) dosimeter has been used since 2008 to replace the dosimetric film (transition started in 2006 and completed in 2008), and, at EDF, the Landauer© OSL "InLight" dosimeter was used since 2006, replacing the Kodak© "type 2" film dosimeters. The most recent dosimeters (RPL and OSL) were considered sufficiently pre-calibrated not to require any correction. The dosimeter used by Orano laboratories has not changed between 2004 and 2014 and remains the thermoluminescent (TLD) dosimeter or TLD "Cogebadge". Consequently, assuming the exposure characteristics at workstations have not changed from 2004, the dose conversion coefficients calculated for the year 2004 for CEA (resp., 2003 for EDF) were retained up to and including 2007 (resp., 2005). From 2008 (respectively, 2006), it was considered not necessary to correct the doses for CEA (resp., EDF) facilities. For Orano, the 2004 coefficients were retained for the duration of the update (2005–2014). Using these correction coefficients, annual recorded doses were converted to personal penetrating photon dose Hp(10) expressed in Sv, as well as absorbed doses expressed in Gray (Gy) to the organs of interest for this study (lung, colon, breast, etc.). Analyses of non-cancer outcomes were conducted based on Hp(10) values in Sv because the target tissue for many non-cancer diseases is not clear yet. Since this study mainly deals with photon radiation, with a radiation-weighting factor of 1, the results could also be expressed in terms of absorbed dose to organs in Gray with similar numerical values [12].

A limited proportion of workers was exposed to neutrons. However, available information on neutron doses was too sparse and uncertain to include neutron doses in the calculation of external doses [31]. Instead, a time-dependent flag was created, labelling the workers as “not exposed” as long as their cumulative neutron dose was null, “weakly exposed” if their cumulative neutron dose was positive but less than 10% of their cumulative total external dose, and “substantially exposed to neutrons” if their cumulative neutron dose exceeded 10% of their cumulative total external dose.

Some workers may have incorporated various radionuclides depending on their activities. However, individual estimates of internal doses from radionuclide intakes are not available for all the cohort members yet since data on internal contamination in occupational settings have only recently been systematically collected and centralized in France. A time-dependent flag was instead created, labelling the workers as “not exposed”, “possibly exposed”, and “exposed”, relying on a work-station exposure matrix and sparsely available bioassays results [32].

### 2.4. Mortality Analysis (Comparison with External Reference)

Mortality in the SELTINE cohort was compared to that of the French general population by calculating standardized mortality ratios (SMR) using national mortality rates as the external reference. An SMR is the ratio of the number of observed deaths in the cohort to the number of “expected” deaths under the hypothesis that mortality rates are the same in the cohort than in the general population. The SMRs were stratified by calendar period in nine categories (1968/1973/1978/1983/1988/1993/1998/2003/2008+), sex, and attained age in 5-year intervals (<15/20/25/…/80/85/90/95+). Byar’s approximation [33] was used to estimate 95% confidence intervals (CI) for the SMRs. Results are presented only for causes of death for which at least 10 deaths were observed.

### 2.5. Analysis of the Dose–Risk Associations (with Internal Reference)

Poisson regression methods were used to estimate the dose–risk relationships between cumulative dose of IR and death from cancer and non-cancerous diseases in the SELTINE cohort (with internal reference). Causes of death for which less than 30 cases occurred were not investigated to avoid obtaining strongly uncertain risk estimations. As male workers represented 87% of the cohort, and as women generally accumulated very low doses, dose–risk analyses were mainly detailed in males. We used linear excess relative rate (ERR) models to describe dose–risk relationships, but departure from linearity was also tested by fitting linear-quadratic models. The linear ERR model was written as λ0(z)[1+βd], where λ0(z) is the background mortality rate depending on z, a set of covariates specific to the disease outcome (including age, calendar year, company, duration of employment, and socioeconomic status), d is the cumulative dose, and β is the estimate of the ERR per unit dose (ERR/Sv or ERR/Gy; see below). Effect modification of the dose–risk relationships by age at exposure and attained age was tested in the analyses of solid cancer and leukaemia excluding chronic lymphocytic leukaemia (CLL, generally thought to be non-related to ionizing radiation). Analyses used the absorbed dose to the organ of interest or the dose equivalent Hp(10). To allow for a latent period in radiation effect, cumulative dose was lagged by 10 years for solid cancers and non-cancerous diseases and by 2 years for leukaemia [12]. These latency values were chosen *a priori* to facilitate comparison with previous international nuclear worker studies [12,13,26], the most recent of which reported a better model fit using a 10 y latency assumption than a 5 y one for solid cancer analyses [13].

Sensitivity analyses were conducted to investigate the influence of adjustment for the neutron flag or the internal contamination potential flag. Analyses were also restricted to workers first employed after 1956 because the dosimeters used before 1957 were dosimeters without filters that were likely to overestimate the response at low energies; doses estimated before 1957 are certainly subject to larger measurement errors than those estimated since the dosimeters with filters were used. Coding of medical causes of death is generally suspected to be less accurate at oldest ages: analyses were further restricted to workers aged less than 80 years at the end of the follow-up to investigate the sensitivity of the findings.

We report likelihood-based 95% CI for the ERRs. All analyses were performed using the SAS® 9.4 and EPICURE [34] software.

This study complies with French ethics recommendations on the use of individual health data and has been approved by the French Data Protection Authority (Comité National de l’Informatique et des Libertés, authorization DR-2012-611).

## 3. Results

### 3.1. Study Population

The study population includes 80,348 workers. In total, 69,487 (86.5%) were male and 10,861 (13.5%) were female workers (Table 1). Altogether, they accrued about 2.55 million person-years of follow-up. About 86% of these person-years were accumulated by male workers. The mean age at hiring was 26 years old for both males and females. The mean duration of employment was 26 years, and only slightly lower in female than in male workers (Table 1). More than half the workers had CEA as their main employer (55.5%) vs. 31.6%, 9.8%, and 3.1% for EDF, Orano, and other (subsidiaries), respectively.

The mean duration of follow-up was 32 years. The mean ages at the beginning and end of follow-up were 31 and 63 years old, respectively. These figures are similar in male and female workers. At the end of follow-up, 15,695 (19.5%) workers had died (20.5% of male and 14.4% of female workers). Only 0.5% of workers were lost to follow-up.

**Figure 1:**
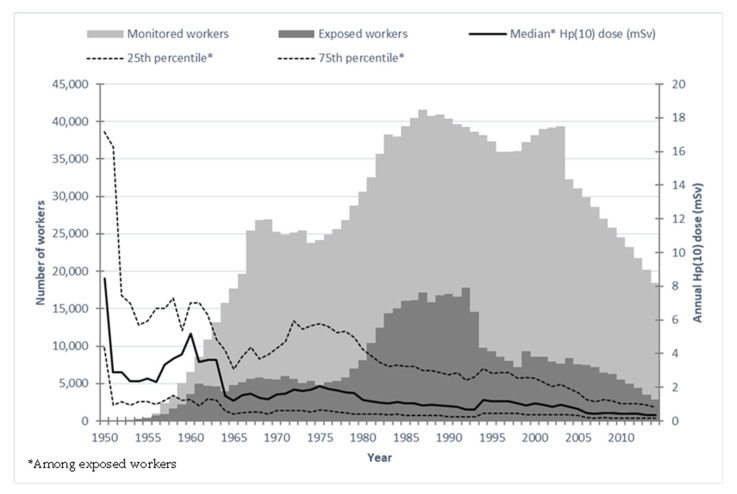
Distribution of monitored and exposed workers with median Hp(10) dose in milliSievert (mSv) by calendar year in SELTINE.

The mean external dose accumulated by workers in Hp(10) quantity was 15.7 mSv (standard deviation: 37.5 mSv) but with a strong gender difference (17.7 mSv in male vs. 3.1 mSv in female workers). The dose distribution was highly skewed, as shown in Table 1. The maximum cumulative dose in Hp(10) was 668.6 mSv. In total, 25,768 workers (32%) received no dose, recorded as “0 mSv”. Figure 1 shows the temporal distribution of dose deposition among male exposed workers but also among the population of male workers as a whole and also the number of these workers over time. About 2% and 0.5% of person-years were flagged with potential for neutron exposure or internal contamination, respectively. 

**Table 1:** Characteristics of the French nuclear worker cohort SELTINE, followed-up for mortality over period 1968–2014.

Characteristics	Male		Female		Total	
Number of workers	69,487		10,861		80,348	
Number of person-years	2,202,492		352,062		2,554,554	
Median year of birth (range)	1951 (1893–1984)		1947 (1896–1983)		1951 (1893–1984)	
Follow-up (1968–2014), in years (SD)						
Mean duration	31.7	(11.4)	32.4	(13.0)	31.8	(11.6)
Mean age at beginning of follow-up	31.1	(8.1)	31.0	(8.8)	31.1	(8.2)
Mean age at end of follow-up	62.8	(13.6)	63.4	(15.3)	62.9	(13.9)
Vital status on 31 December 2014, n (%)						
Alive	55,134	(79.3)	9164	(84.4)	64,298	(80.0)
Deceased	14,131	(20.4)	1564	(14.4)	15,695	(19.5)
Lost to follow-up	222	(0.3)	133	(1.2)	355	(0.5)
Employment						
Median year of hiring (range)	1977	1938–2004	1974	1940–2003	1977	1938–2004
Mean age at hiring, in years (SD)	26.4	(6.3)	26.1	(7.0)	26.3	(6.4)
Mean duration, in years (SD)	26.5	(9.0)	24.4	(10.6)	26.2	(9.2)
Hired after 1956, n (%)	66,003	(95.0)	10,239	(94.3)	76,242	(94.9)
Number of person-years by principal * employing company (%)						
CEA	1,133,865	(51.5)	284,956	(80.9)	1,418,821	(55.5)
Orano	232,415	(10.5)	17,247	(4.9)	249,662	(9.8)
EDF	768,889	(34.9)	39,299	(11.2)	808,188	(31.6)
Other **	67,323	(3.1)	10,560	(3.0)	77,883	(3.1)
Socioeconomic status, n (%)						
Managers and engineers	13,466	(19.4)	2492	(22.9)	15,958	(19.9)
Administrative employees	2909	(4.2)	3952	(36.4)	6861	(8.5)
Skilled workers	40,537	(58.3)	3448	(31.8)	43,985	(54.7)
Unskilled workers	11,787	(17.0)	827	(7.6)	12,614	(15.7)
Unknown	788	(1.1)	142	(1.3)	930	(1.2)
Monitoring of external radiation exposure, in years						
Mean duration (SD)	21.2	(9.9)	15.0	(9.8)	20.3	(10.1)
Mean age at last monitoring (SD)	50.0	(9.7)	43.7	(11.5)	49.2	(10.2)
Number of exposed *** workers (%)	49,995	(71.9)	4585	(42.2)	54,580	(67.9)
Cumulative Hp(10) dose, in milliSieverts (SD)						
Mean (SD), whole cohort	17.7	(39.7)	3.1	(13.7)	15.7	(37.5)
Median/IQR/maximum, whole cohort	2.1/0.0–16.1/668.6		0.0/0.0–0.9/573.9		1.3/0.0–13.0/668.6	
Mean (SD), exposed *** workers only	24.7	(44.9)	7.3	(20.4)	23.1	(43.6)
Person-years by categories of 10-years lagged cumulative Hp(10) dose in milliSievert, n (%)						
<2.5	1,487,620	(67.6)	308,589	(87.7)	1,796,209	(70.3)
2.5 –4.9	128,096	(5.8)	13,295	(3.8)	141,391	(5.5)
5.0 –9.9	135,109	(6.1)	10,724	(3.0)	145,833	(5.7)
10.0–19.9	142,935	(6.5)	9470	(2.7)	152,405	(6.0)
20.0–49.9	169,571	(7.7)	7281	(2.0)	176,852	(6.9)
50.0–99.9	85,740	(3.9)	1865	(0.5)	87,605	(3.4)
100.0–199.9	42,162	(1.9)	641	(0.2)	42,803	(1.7)
≥200.0	11,259	(0.5)	197	(0.1)	11,456	(0.5)
Person-years by status of neutron exposure, n (%)						
Not exposed	2,159,095	(98.0)	349,872	(99.4)	2,508,967	(98.2)
Potentially exposed	43,397	(2.0)	2190	(0.6)	45,587	(1.8)
Person-years by status of potential for internal contamination, n (%)						
No	2,127,408	(96.6)	340,711	(96.8)	2,468,119	(96.6)
Low/medium potential	63,372	(2.9)	8780	(2.5)	72,152	(2.8)
High potential	11,712	(0.5)	2571	(0.7)	14,283	(0.6)

SD: standard deviation; IQR: interquartile range; * company in which the employee was hired for the longest period of time and, in case of a tie, the company corresponding to the first hiring is retained; ** mainly from subsidiaries of Orano; *** with at least one positive recorded dose.

### 3.2. Mortality Analysis (Comparison with External Reference)

Supplementary Table S1 shows the distribution of causes of death in the full cohort over the 1968–2014 period. In total, 6236 of the 15,695 deaths (40%) were due to cancer, among which 5691 (36%) were due to solid cancers. Further, 545 deaths (3.5%) were due to tumours of lymphatic and hematopoietic tissues. Among non-cancerous causes of death, circulatory diseases were the leading causes, with 3582 deaths (22.8% of all deaths).

Supplementary Table S1 reports SMRs for each cause of death. Overall, a strong healthy worker effect (HWE) was observed in the cohort, as reflected by a significant mortality deficit by comparison with the French general population. This is observed for deaths as a whole (SMR = 0.64; 95% CI: 0.63–0.65) but also for deaths by all solid cancers combined (SMR = 0.71; 95% CI: 0.69–0.73). This is also statistically significant but of lesser magnitude for tumours of lymphatic and hematopoietic tissues combined (SMR = 0.90; 95% CI: 0.83–0.98) and not significant for leukaemia excluding CLL (Supplementary Table S1). Only mortality by pleural cancer showed a statistically significant excess (SMR = 1.68; 95% CI: 1.36–2.05). An excess of death by skin melanoma was close to statistical significance (SMR = 1.22; 95% CI: 0.99–1.50). A few other non-significant excesses were observed for breast (in women), ovary, brain, and central nervous system cancers, and for acute lymphoblastic leukaemia. No excess was observed for deaths by non-cancerous diseases (Supplementary Table S1).

### 3.3. Analysis of the Dose–Risk Associations (with Internal Reference)

Because of the small proportion of women in the cohort and their overall very low cumulative doses, results of analyses of the dose–risk associations will be presented mainly in males for different outcomes.

#### 3.3.1. Solid Cancers

**Figure 2:**
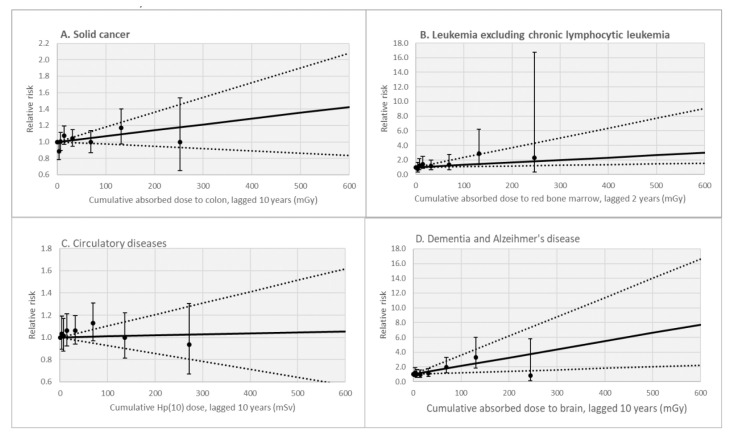
Relative risk of mortality due to solid cancer (panel **A**), leukaemia excluding chronic lymphocytic leukaemia (panel **B**), circulatory diseases (panel **C**), and dementia and Alzheimer’s disease (panel **D**) by categories of cumulative dose in milliGray (mGy) in male workers, SELTINE. Solid line: fitted linear dose–risk model (at attained age 65 years for panel B); dashed lines: 95% confidence interval; dark circles and associated vertical lines: point estimates of relative risks and associated 95% confidence interval.

Table 2 reports parameter estimates of ERR models for mortality by cancer for a 10-year lagged cumulative colon dose in males only. A non-statistically significant positive dose–risk relationship was observed for the group of solid cancers combined based on a simple linear ERR model (ERR/Gy = 0.71; 95% CI: −0.28; 1.80) (Figure 2). Similar results were observed for the whole cohort (including females), with a non-statistically significant positive dose–risk relationship observed for the group of solid cancer based on a simple linear ERR model (ERR/Gy = 0.69; 95% CI: −0.28; 1.77).

In male workers, the linear model better described the data than a linear quadratic model (see Supplementary Table S2 for comparison of Akaike information criterion (AIC) by model: a lower AIC indicates a better fit to the data). Introduction of effect modifiers for age at exposure or attained age did not improve the fit either, indicating no substantial modification of effect by these factors. When study of the dose–risk relationship was restricted to workers hired after year 1956, the value of the ERR remained similar (ERR/Gy = 0.65; 95% CI: −0.40; 1.83). When analyses were restricted to the dose range of 0–100 mGy, the ERR estimate decreased (ERR/Gy = 0.57; 95% CI: −1.14; 2.41) and the *p*-value increased. Adjustment for potential exposure to neutrons or internal contamination did not markedly change the ERR estimate and did not improve the model fit. Excluding years of follow-up from age 80 years led to a slight decrease in estimated ERR/Gy = 0.59 (95%CI: −0.49; 1.76; *n* = 4361).

Table 2 shows ERR estimates for different site-specific solid cancers in relation with doses to specific target organs or tissues. When analysing the grouping of causes of death “solid cancers excluding lung cancers”, the value of the ERR was slightly diminished by comparison with the ERR obtained for all solid cancers (Table 2). It remained positive but not statistically significant (ERR/Gy = 0.45; 95% CI: −0.65; 1.69). The results are overall imprecise, and, for many specific cancer sites, lower bounds of 95% Cis could not be estimated due to a lack of convergence. However, the ERR estimates were not statistically significant for any specific cancer site, as indicated by the *p*-values.

**Table 2:** Excess relative rates * of death from solid cancers (including specific cancer sites) per Gray (ERR/Gy) of 10-year lagged cumulative dose absorbed to target tissues in males only, SELTINE cohort, period 1968–2014.

Cause of Death	Target Tissue	Observed Deaths	ERR/Gy	95% CI	*p*-Value
All cancers	Colon	5,618	0.53	−0.40; 1.55	0.28
All cancers excluding leukaemia	Colon	5,414	0.46	−0.48; 1.50	0.35
Solid cancers	Colon	5,130	0.71	−0.28; 1.80	0.16
Solid cancers excluding lung cancer	Colon	3,778	0.45	−0.65; 1.69	0.44
Oral cavity cancer	Skin	201	4.54	−0.92; 12.8	0.12
Oesophagus cancer	Stomach	206	1.26	ne; 7.97	0.63
Stomach cancer	Stomach	198	2.82	ne; 10.7	0.32
Colon cancer	Colon	383	1.48	−1.71; 6.03	0.42
Rectum cancer	Colon	142	4.31	−1.24; 13.1	0.15
Liver cancer	Liver	286	1.20	ne; 7.17	0.63
Gallbladder cancer	Gallbladder	35	NC	NC	NC
Pancreas cancer	Pancreas	338	−2.41	ne; 1.19	0.15
Peritoneum cancer	Colon	74	6.58	ne; 23.9	0.18
Nasal cancer	Skin	45	8.87	ne; 33.5	0.15
Larynx cancer	Stomach	102	5.23	ne; 18.6	0.22
Lung cancer	Lung	1,352	1.09	−0.83; 3.39	0.29
Pleural cancer	Lung	95	−1.79	−2.14; 7.03	0.61
Bones, connective, and other soft tissues cancers	Colon	48	3.67	ne; 22.8	0.52
Melanoma	Skin	86	3.68	ne; 17.0	0.37
Prostate cancer	Bladder	473	−1.66	ne; 1.42	0.25
Bladder cancer	Bladder	212	−1.14 **	ne; 4.63	0.65
Kidney cancer	Bladder	160	3.59	ne; 13.8	0.32
Brain and central nervous system cancer	Brain	169	−1.68	ne; 5.31	0.56
Brain and central nervous system tumours including benign tumours	Brain	250	0.87	ne; 7.71	0.75

* estimated from a linear model of cumulative penetrating photon dose lagged by 10 years, adjusted on calendar year, age, company, duration of employment, and socioeconomic status (except for bones, melanoma, and brain tumours); ** categories of cumulative dose above 20 mGy were collapsed; CI: likelihood-based confidence interval; *p*-value of a likelihood ratio test vs. no effect of dose; NC: convergence not achieved; ne: not estimated.

#### 3.3.2. Hematopoietic and Lymphatic Cancers

Supplementary Table S3 reports parameter estimates of ERRs for death from leukaemia excluding CLL, for 2-year lagged cumulative red bone marrow doses, in males. The estimate from a simple linear ERR model was positive and statistically significant (ERR/Gy = 9.49; 95% CI: 1.60; 21.36). However, AIC values indicate that the fit was better for a model introducing attained age as an effect modifier (ERR/Gy = 3.31 at age 65; 95% CI: 0.94; 13.38). In contrast, introducing age at exposure as an effect modifier did not improve the fit. If the years of follow-up from age 80 onward were excluded, there was no longer a modifying effect of attained age and the dose–risk association was no longer statistically significant, based on 126 deaths (ERR/Gy = 4.55 (95%CI: <−3.03; 15.61). In the whole cohort (including females), a statistically significant positive dose–risk relationship was observed for the group of leukaemia excluding CLL based on a simple linear ERR model (ERR/Gy = 3.65 at age 65; 95% CI: 1.25; 13.88) (Figure 2).

The association observed in male workers remained significant when the study population was restricted to workers hired after year 1956. When analyses were restricted to a dose range of 0–100 mGy, the ERR estimate remained close to statistical significance (ERR/Gy = 1.37 at age 65; 95% CI: 0.06; 16.19), but the *p*-value was 0.09, therefore above the conventional value of 0.05 for statistical significance. Adjustment for potential exposure to neutrons or internal contaminations had limited impacts on ERR values.

Myelodysplastic syndrome (MDS, ICD-10: D46)), a preleukemic condition that mainly affects adults of advanced age (median age of about 80 years), can develop into acute myeloid leukaemia; until the mid-1980s, cases were not identified as MDS and were often misdiagnosed as acute myeloid leukaemia. Including MDS in the acute myeloid leukaemia subgroup decreased the estimated ERR/Gy but improved the accuracy of the estimation (Table 3). Models did not converge for non-Hodgkin lymphoma, and no association with dose was observed for multiple myeloma in males only (Table 3).

#### 3.3.3. Non-Cancer Diseases

As shown in Table 4, IR exposure was not associated with death from all non-cancer diseases as a group (ERR/Sv = 0.02; 95% CI: −0.49; 0.57). Considering specific diseases one at a time, the only statistically significant increase in risk was observed for death by dementia, including Alzheimer’s disease (ERR/Sv = 9.62; 95% CI: 3.05; 18.68). Figure 2 shows the dose-category-specific relative risk estimate for this cause of death. ERRs were elevated but not statistically significant for several diseases, e.g., cerebrovascular diseases and hypertensive (but not ischemic) diseases, cirrhosis. For mortality by cerebrovascular diseases, when the absorbed dose to the brain was considered instead of the Hp(10) dose, the estimate of the ERR/Gy remained not statistically significant (ERR/Gy= 2.56; 95% CI: −0.81; 6.93; *p* = 0.17). Similar results were observed in the whole cohort, including female workers (results not shown). No association was observed with death from all circulatory diseases (Figure 2), chronic obstructive pulmonary diseases (COPD), or other respiratory or digestive diseases.

Excluding the years of follow-up from age 80 onward increased risk estimates for mental disorders (ERR/Gy = 7.19; 95%CI: −0.81; 20.52; *p* = 0.09) and diseases of the nervous system (ERR/Gy = 5.38; 95%CI: 0.21; 12.42; *p* = 0.04), notably of Parkinson disease (ERR/Gy = 5.88; 95%CI: <−5.28; 25.93; *p* = 0.33). The risk estimate for mortality by dementia and Alzheimer’s disease was slightly increased (ERR/Gy = 11.21 (95%CI: 2.02; 25.98), *p* = 0.01, based on 105 deaths). The results for circulatory diseases were also modified as the estimated ERR/Sv was equal to 0.76 (95%CI: −0.30; 1.99; *p* = 0.17, based on 2262 deaths); for ischemic diseases, the estimated ERR/Sv increased to 0.65 (95%CI: −1.01; 2.71; *p* = 0.473), while it was more stable for cerebrovascular diseases (ERR/Sv = 1.84; 95%CI: −0.72; 5.30; *p* = 0.182).

**Table 4:** Excess relative rates * (ERR) of death from non-cancer diseases per unit (in Gray or Sievert) of 10-year lagged cumulative dose absorbed to target tissues (in Gy or Sv) in males only, SELTINE cohort, period 1968–2014.

Cause of Death	Target Tissue	Observed Deaths	ERR/Gy or ERR/Sv	95% CI	*p*-Value
Non-cancer diseases (all)	H_p_(10)	8577	0.02	−0.49; 0.57	0.95
Diabetes mellitus	H_p_(10)	169	−0.91	ne; 2.62	0.54
Mental and behavioural disorders	Brain	235	2.74	−1.95; 10.33	0.32
Diseases of the nervous system	Brain	531	3.47	−0.48; 8.56	0.09
Dementia, Alzheimer’s disease, Parkinson’s disease, motoneuron disease	Brain	469	4.99	0.74; 10.52	0.02
Dementia, Alzheimer’s disease	Brain	269	9.62	3.05; 18.68	<0.01
Parkinson’s disease	Brain	124	−1.30	ne; 7.44	0.71
Circulatory diseases	H_p_(10)	3261	0.09	−0.72; 1.03	0.83
Ischemic diseases	H_p_(10)	1258	−0.23	ne; 1.38	0.76
Cerebrovascular diseases	H_p_(10)	684	1.41	ne; 4.05	0.19
Hypertensive diseases	H_p_(10)	120	2.31	ne; 9.98	0.37
Respiratory diseases	Lung	558	0.22	ne; 3.71	0.88
Chronic obstructive pulmonary diseases	Lung	164	0.15	ne; 7.52	0.96
Digestive diseases	Colon	583	0.96	−1.72; 4.58	0.53
Cirrhosis	Liver	166	3.72	ne; 13.64	0.29

* estimated from a linear model of cumulative penetrating photon dose lagged by 10 years, adjusted on calendar year, age, sex, company, duration of employment, and socioeconomic status; CI: likelihood-based confidence interval; ne: not estimated.

## 4. Discussion

In this analysis of the updated and extended French cohort of nuclear workers monitored for external exposure to IR, a strong healthy worker effect was observed overall, but death from pleural cancer was in significant excess. Death from solid cancers was positively but non-significantly associated with IR exposure. Death from leukaemia (excluding CLL) was positively and significantly associated with IR exposure. No association between cumulative dose and risk of mortality from non-cancerous diseases was observed overall, except a statistically significant dose–risk relationship for death by dementia and Alzheimer’s disease.

### 4.1. General Strengths and Limitations 

The major strengths of the cohort are the availability of individual external dosimetry data, the professional stability of the population, a long duration of follow-up, and a very low percentage of people lost to follow-up. A limitation is the lack of individual information about classical lifestyle risk factors for cancer and non-cancer diseases, such as smoking. However, the lack of association between dose and death by COPD suggests the absence of any substantial bias from smoking. In addition, a quite detailed indicator on socioeconomic status is available, which indirectly reflects major disparities in exposure to unmeasured risk factors (and resulting baseline risks of death) across socioeconomic positions. As explained above, dose estimates before 1956 were surrounded with specific uncertainties, which is often the case for most archaic dose estimates in other nuclear workers studies [35,36]. However, when analyses were restricted to workers first employed after 1956, the estimates of ERR/Gy remained similar.

As part of the current analysis, investigations of the shape of dose–risk relationships or different lag times were not conducted in extensive detail. This is because forthcoming updated INWORKS analyses, which will include the updated SELTINE cohort data, will have a much better capacity to investigate these aspects. Finally, the current analysis was limited to mortality because of the lack of nationwide registries in France to track the incidence of cancer and non-cancer chronic disease. 

### 4.2. Mortality Analyses (Comparison with External Reference)

Overall, the mortality rates in the SELTINE cohort are much lower than in the French general population. Such patterns are classically observed in cohorts of workers, including cohorts of radiation workers [37,38]. This does not mean that IR has a protective effect. Instead, this observation likely results from the HWE, which is due to complex selection effects of people sufficiently healthy to be hired and to keep their job for a long time after hiring (here, for at least 1 year, but the mean length of employment is 26 years) [39]. In addition, there are likely positive effects of regular follow-up health checks by occupational physicians. The HWE tends to shrink as populations get older, notably after retirement age [40]. For instance, the SMR for all causes was 0.60 (95%CI 0.59–0.62) as part of the previous analysis of the cohort followed-up until year 2004 [24] and has now slightly increased to 0.64 (95%CI 0.59–0.62) with a follow-up extended until year 2014.

Mortality from pleural cancer was already observed in previous analyses of the cohort and remains very stable: SMR = 1.69; 95%CI: 1.22–2.27 based on a follow-up until the end of year 2004 [24] vs. SMR = 1.68; 95%CI: 1.36-2.05 based on a follow-up until the end of year 2014 in the present analysis. This cause of death was not associated with radiation exposure in dose–risk analyses (see Table 2) and is generally not associated with IR [41,42], with few exceptions [18]. Exposure to asbestos is a likely causal agent for excesses of pleural cancers in industrial settings, including in nuclear facilities [42].

### 4.3. Dose–Risk Relationships (with Internal Reference)

#### 4.3.1. Solid Cancers

Although we observed no statistically significant association between radiation exposure and solid cancers in the current analysis, possibly because of insufficient statistical power, the magnitude of our estimated ERR/Gy (0.71; 95% CI: −0.28; 1.80) is compatible with the (statistically significant) estimate obtained in INWORKS including 308,297 workers (ERR/Gy = 0.47; 90% CI: 0.18; 0.79) [13]. Direct comparison of our results with the latest results from the atomic bomb survivor study is complicated because of different population characteristics (e.g., age and sex distribution differ in the full cohorts) [3]. A recent comparison of radiation-related risk of death by solid cancers in INWORKS and in comparable subjects from the A-bomb survivor study (subsets of, respectively, 259,350 from INWORKS and 45,625 atomic bomb survivors selected to improve comparability of the cohorts with respect to age, periods of exposure, and of follow-up) revealed very close estimates of ERR/Gy for solid cancer [43]. This indirectly suggests compatibility of our findings with those from the atomic bomb survivor study since our estimate of ERR/Gy for solid cancer is compatible with that from INWORKS.

A recent analysis of solid cancer incidence in a British cohort of 172,452 radiation workers also reported a similar statistically significant estimate ERR/Sv= 0.52, 95% CI: 0.11; 0.96) [18]. In a Japanese cohort of 204,103 nuclear power plant workers, the ERR/Gy was 1.22 (90% CI: 0.24, 2.26) for all cancers excluding leukaemia [44]. By contrast, in a recent study of 135,193 US nuclear power plant workers included in the larger US Million Person Study, the ERR per Gy (95% CI) for mortality by solid cancers was 0.1 (−0.3; 0.5) [45]. This estimate is much lower but still statistically compatible with that from the SELTINE cohort. Analyses from other subgroups of the Million Person Study should be published soon and allow for a more detailed comparison with other occupationally exposed groups. Overall, our results for solid cancers appear to be broadly similar to, or at least compatible with, those observed in other major cohorts of radiation workers.

#### 4.3.2. Leukaemia Excluding CLL

The estimate of ERR/Gy for leukaemia excluding CLL for a simple linear model (without age as an effect modifier) is consistent but more precise and higher than that estimated in the previous analysis of the cohort followed-up until year 2004, which was not statistically significant [24]. It is also compatible with, but higher than, estimates obtained in INWORKS [26], which were themselves comparable to those obtained in the A-Bomb survivor study [43]. However, in the current analysis, the model best fitted to the data including attained age as an effect modifier. The value of the ERR/Gy in this model was then very close to those observed in the previously cited studies [26,43] and in the latest analysis of the United Kingdom Radiation Registry of Radiation Workers [46]. In US NPP workers, the ERR per 100 mGy for leukaemia other than CLL was slightly lower (0.15; 90% CI: −0.001; 0.31) [45], whereas, in Japanese NPP workers, it was negative but with very large statistical uncertainty (ERR/Gy = −0.42; 90%CI: −5.38, 7.59); therefore, these results remain statistically compatible with our findings [44].

#### 4.3.3. Circulatory Diseases

Risks of death by circulatory diseases were not significantly associated with IR exposure in the SELTINE cohort. The risk coefficient was negative for ischemic diseases but positive for cerebrovascular and hypertensive diseases. In INWORKS, the association with mortality from cerebrovascular disease was also higher than that due to mortality due to ischemic heart disease, but then both associations were positive and statistically significant (ERR/Sv = 0.50; 90% CI: 0.12, 0.94 and ERR/Sv = 0.18; 90% CI: 0.004, 0.36, respectively) [7]. In the latest analyses of the British cohort of nuclear workers, mortality from heart diseases [47] and cerebrovascular diseases [48] were both significantly associated with IR exposure. A nested matched case-control study of ischemic heart diseases within the subset of British Nuclear Fuel Cycle Workers investigated potential confounding by lifestyle, physiological traits, and occupational exposures [49]. These analyses found little impact of adjustment for these potential confounders, except possibly for occupational noise exposure [49]. Other case-control studies on mortality by circulatory diseases in uranium plant workers [50] and miners [51] that explored the impact of adjustment for individual risk factors documented in occupational medicine files reached similar conclusions, as well as the study of Mayak PA workers, in which the studied dose range was, however, much higher than in the other workers studies mentioned above [17]. Conversely, no significant association between external radiation and mortality from circulatory diseases was observed in other large cohorts of radiation workers, such as German uranium miners [52] or US NPP workers [45], although, in the latter, dose–risk analyses have only been published for heart diseases so far. Further studies are, therefore, necessary to better characterize and interpret associations between low-dose radiation and circulatory diseases [9].

#### 4.3.4. Dementia

Dose–risk analyses for mortality by dementia had not been analysed as part of the previous follow-up of the cohort [24] and have rarely been conducted in other cohorts of nuclear workers. Figure 2 shows that this result is not driven by excess risk in the highest dose category. Dementia and Alzheimer’s disease often occur in elderly people: in SELTINE, deaths due to dementia and Alzheimer’s disease represent 0.99% of deaths in the 1968–2004 period but 3.59% of deaths from 2005 through 2014, with an average age at death of 82 years versus 68 years for people who died from another cause. However, restricting the follow-up time to a maximum of 80 years old did not reduce, and actually slightly increased, the estimates of ERR/Gy for this outcome. 

An early nested case-control study in a cohort of American female nuclear weapons workers reported significant trends of increasing odds ratios of death by dementia across increasing dose categories based on 91 cases and 910 controls [53]. In this study, this significant finding was driven by a small number of exposed cases.

In INWORKS, mortality by mental disorders was associated with accumulated IR doses (ERR/Sv = 1.30; 90% CI: 0.23, 2.72) based on 705 deaths. Mortality by dementia accounted for 53% of the 705 deaths by mental disorders, but no specific analysis for mortality by dementia was conducted [7]. Among atomic bomb survivors exposed at or after adolescence, radiation did not significantly affect cognition [54] or dementia incidence based on the latest available follow-up [55].

A recent literature review and meta-analysis of epidemiological studies on non-cancerous diseases of the central nervous system in people exposed to low-to-moderate doses of IR during adulthood reported increased risk of Parkinson’s disease associated with low dose exposure, but no such estimate was produced for dementia/Alzheimer’s disease, possibly because of more heterogeneous definitions for this outcome in the reviewed studies [11]. 

The biological mechanisms behind radiation-induced cognitive effects are not fully clear yet [10]. However, several plausible pathways have been proposed: for instance, implying radiation-induced neuroinflammation [10] or oxidative stress and its influence on ageing [56].

There is a possibility that some unmeasured risk factors might have contributed to the association observed between IR exposure and mortality by dementia (confounding bias). The risk factors for dementia are not exhaustively known, but many are common with circulatory diseases, e.g., tobacco consumption, diet, physical activity, alcohol use, overweight/obesity, hypertension, diabetes mellitus, dyslipidaemia [57], and information on these risk factors should be available in occupational medicine files. Whether these risk factors are associated with occupational exposure to IR (at each given level of socioeconomic status reflected by our detailed job classification) is not likely, but this possibility cannot be formally excluded either. Other cohorts or nested case-control studies with individual information on these risk factors would be helpful to explore this hypothesis.

Comparison of our results with those from more studies would be useful to appreciate if the observed associations in this and in two other studies [7,53] remain relatively isolated or can be replicated. Further analyses of dementia in other low-dose cohorts, including updated analyses of pooled cohorts in INWORKS, will be very important to assess whether this association would be consistent across populations.

### 4.4. Perspectives

At the end of year 2014, corresponding to the current end of the follow-up period of the SELTINE cohort, 80% of the workers were still alive. Further follow-up of the cohort is, therefore, warranted to express its full information potential in the future. Before that time, inclusion of the cohort in pooled analyses will contribute to increase the power of statistical analyses. This will be conducted as part of the continuation of INWORKS, which is one of the most informative low-dose IR studies about the dose–risk relationships for death by cancers [4,5] but also by non-cancer diseases.

## 5. Conclusions

The long follow-up time accrued by the SELTINE cohort makes it informative with respect to the long-term risks following protracted exposure to low-dose IR on mortality from both cancer and non-cancer diseases. A strong healthy worker effect was observed overall. The estimated dose–risk relationships were consistent with those from other nuclear worker studies for all solid cancers and leukaemia excluding CLL but remained associated with large uncertainty. Statistically significant dose–risk relationships were observed for mortality by leukaemia and dementia. At this stage, the observed association between dementia mortality risk and low-dose IR should be interpreted with caution and calls for replication by other low-dose studies. This study is the most informative one ever conducted in France among nuclear workers. Participation in future pooled analyses of cohorts of nuclear workers and continuing follow-up will provide further insights into the nature of dose–risk relationships for cancer and non-cancer effects.

## Figures and Tables

**Table 3:** Excess relative rates * of hematopoietic and lymphatic cancer death per Gray (ERR/Gy) of cumulative red bone marrow dose in males only, SELTINE cohort, period 1968–2014.

Cause of Death	Observed Deaths	Lag	ERR/Gy	95% CI	*p*-Value
Leukaemia excluding CLL	157	2	3.31	0.94; 13.38	<0.01
Acute myeloid leukaemia	59	2	5.26	ne; 24.17	0.05
Acute myeloid leukaemia and MDS	96	2	3.86	0.43; 21.36	0.02
Leukaemia (excluding CLL) and MDS	194	2	2.87	0.95; 11.55	0.01
Chronic lymphocytic leukaemia	44	10	NC	NC	NC
Non-Hodgkin lymphoma	184	10	NC	NC	NC
Multiple myeloma	80	10	−0.57	ne; 11.94	0.90

* estimated from a linear model of cumulative penetrating photon dose lagged by 2 or 10 years, adjusted on calendar year, age, company, and duration of employment, and allowing for modification effect of attained age for leukaemia analyses; CI: likelihood-based confidence interval; CLL: chronic lymphocytic leukaemia; MDS: myelodysplastic syndromes; NC: convergence not achieved; ne: not estimated.

## Data Availability

The study’s data are not publicly available since they are individual and sensitive data partly obtained from CEA, EDF, and Orano. They might become available for specific research projects from the authors with the permission of IRSN, CEA, EDF, and Orano. Proposals for collaborations in further analyses of the data should be addressed to the following email address: seltine@irsn.fr.

## Supplementary Materials

The following supporting information can be downloaded at: https://www.mdpi.com/article/10.3390/cancers15010079/s1, Table S1: ICD-10 codes, number of deaths, standardized* mortality ratios (SMR), and 95% confidence intervals (CI) in the SELTINE cohort, period 1968–2014; Table S2: Parameter estimates of the excess relative rate (ERR) models for solid cancer and 10-year lagged cumulative colon dose in males only, SELTINE cohort, period 1968–2014; Table S3: Parameter estimates of the excess relative rate (ERR) models for leukaemia (excluding chronic lymphocytic leukaemia) and 2-year lagged cumulative red bone marrow dose in males only, SELTINE cohort, period 1968–2014.
