# Supplementary material for: Updated Mortality Analysis of SELTINE, the French Cohort of Nuclear Workers, 1968–2014
Source: Cancers (Basel). 2022 Dec 23;15(1):79. doi: 10.3390/cancers15010079 (PMC9817793; doi:10.3390/cancers15010079)
Supplement: Supplementary file 1 [file cancers-15-00079-s001.zip › cancers-2026988-supplementary.pdf]

Supplementary Table S1. ICD-10 codes, number of deaths, standardized* mortality ratios (SMR) and 95% confidence intervals (CI) in the SELTINE cohort, period 1968-2014.

Cause of death	ICD-10 codes	Observed deaths	SMR	95%CI
**All causes**		15,695	0.64	0.63-0.65
**Solid cancers**	C00–C80, C97, except C46.3	5691	0.71	0.69-0.73
Mouth and pharynx	C00–C14, C46.2	206	0.42	0.37-0.49
Oesophagus	C15	211	0.52	0.45-0.60
Stomach	C16	210	0.67	0.58-0.76
Colon	C18	428	0.76	0.69-0.84
Rectum	C19–C21	155	0.69	0.59-0.81
Liver	C22	307	0.62	0.55-0.69
Gallbladder	C23–C24	41	0.77	0.55-1.04
Pancreas	C25	367	0.92	0.82-1.01
Peritoneum	C26, C45.1, C48	84	0.75	0.59-0.92
Nasal cavity	C30–C31	48	0.44	0.32-0.58
Larynx	C32	103	0.44	0.36-0.53
Trachea, bronchus, and lung	C33–C34	1421	0.67	0.64-0.71
Pleura	C38.4, C45.0	98	1.68	1.36-2.05
Bones and articular cartilage	C40–C41	32	0.81	0.56-1.15
Soft tissue	C46.1, C47, C49	19	0.55	0.33-0.86
Skin melanoma	C43	94	1.22	0.99-1.50
Skin other	C44, C46, C46.0, C46.9	15	0.65	0.36-1.07
Breast (women)	C50	153	1.04	0.88-1.22
Corpus uteri (women)	C54–C55, C58	23	0.76	0.48-1.15
Ovary (women)	C56, C57.0–C57.4, C57.8	50	1.14	0.84-1.50
Prostate (men)	C61	473	0.84	0.76-0.92
Bladder	C67, C68	221	0.70	0.61-0.80
Kidney	C64–C66	166	0.85	0.73-0.99
Brain and central nervous system	C70–C72	189	1.05	0.91-1.21
**Tumors of lymphatic and hematopoietic tissues**	C46.3, C81–C96	545	0.90	0.83-0.98
Hodgkin lymphoma	C81	22	0.87	0.55-1.32
Non-Hodgkin lymphoma	C46.3, C82–C85, C88.0, C88.1, C88.3, C91.4, C96	207	0.95	0.82-1.08
Multiple myeloma and IP** tumors	C88, C88.2, C88.7, C88.9, C90	89	0.86	0.69-1.05
Leukemia excluding CLL***	C91.0, C91.2–C91.3, C91.5, C91.7, C92–C95	176	0.87	0.75-1.01
Acute myeloid leukemia	C92.0, C92.4–C92.5, C93.0, C94.0, C94.2–C94.5	67	0.91	0.70-1.15
Chronic myeloid leukemia	C92.1, C93.1, C94.1	22	0.74	0.46-1.12
Acute lymphoblastic leukemia	C91.0	13	1.11	0.59-1.90
Chronic lymphocytic leukemia	C91.1	48	0.99	0.73-1.31
**Non-cancer causes of death**	E10–E14			

Cause of death	ICD-10 codes	Observed deaths	SMR	95%CI
Diabetes mellitus	F00–F99	188	0.49	0.43-0.57
Mental and behavioural disorders	G00–G99	269	0.45	0.39-0.50
Diseases of the nervous system	F00, F01, F03, F05, G30, G20, G12.2	618	0.79	0.73-0.86
Dementia, Alzheimer's disease, Parkinson's disease, moto-neuron disease	F00, F01, F03, F05, G30	553	0.85	0.78-0.92
Dementia and Alzheimer's disease	G20	334	0.81	0.72-0.90
Parkinson's disease	G45, I00–I99	139	0.99	0.83-1.17
Circulatory diseases	I20–I25	3582	0.62	0.60-0.64
Ischemic heart diseases	G45, G45.0–G45.2, G45.8–G45.9, I60–I69	1333	0.66	0.62-0.69
Cerebrovascular diseases	G45, G45.0–G45.2, G45.8–G45.9, I60–I69	781	0.61	0.57-0.66
Respiratory diseases	J00–J99	628	0.48	0.44-0.52
COPD	J40–J44, J47	186	0.43	0.37-0.49
Asthma	J45–J46	28	0.50	0.33-0.72
Digestive diseases	K00–K93	633	0.42	0.39-0.46
Cirrhosis	K70	177	0.32	0.28-0.37
**External causes**	V01–Y89	1559	0.63	0.60-0.66

ICD-10: International Classification of Diseases, 10th revision; *on calendar period, age, and sex; ** immuno-proliferative; *** chronic lymphocytic leukemia.

Supplementary Table S2. Parameter estimates of the excess relative rate (ERR) models for solid cancer and 10-year lagged cumulative colon dose in males only, SELTINE cohort, period 1968-2014.

ERR model[a] (per Gy)	Coefficient	95%CI	AIC	p-value
ERR(*d*)=β*d*	β = 0.71	-0.28; 1.80	11671	0.16[b]
Restricted range of cumulative dose 0-100 mGy (*n*=4986)	β = 0.57	-1.14; 2.41		0.53[c]
First employment after 1956 (*n*=4357)	β = 0.65	-0.40; 1.83		0.23[b]
ERR(*d*)=β*d* +γ*d*²	β = 1.37	-0.68; ne	11672	0.47[c]
	γ = -4.32	ne; 7.94		
ERR(*d*,*aex*)=β*d*·exp(υ·*aex*)	β = 0.68	-0.27; 1.78	11672	0.70[d]
	$e^{υ}$ = 0.69	ne		
ERR(*d*,*a*)=β*d*·$a^{υ}$	β = 0.84	-0.19; 1.97	11672	0.35[d]
	υ = -2.42	ne		
ERR(*d*)=β*d* adjusted on neutron flag	β = 1.00	-0.06; 2.18	11672	0.21[b]
ERR(*d*)=β*d* adjusted on internal contamination flag	β = 0.70	-0.29; 1.80	11674	0.74[b]

[a] defined as $λ_0$(*y*,*a*,*c*,*ses*,*duem*)[1 + ERR(*d*,*cov*)], where *y* is calendar year, *a* is attained age, *c* is company, *ses* is socioeconomic status, *duem* is duration of employment, *d* is cumulative colon dose, and *cov* is either attained age (*a*) or age at exposure (*aex*); CI: likelihood-based confidence interval; AIC: Akaike information criteria; p-value of a likelihood ratio test vs. [b] tested hypothesis: β=0, [c] tested hypothesis: γ=0, [d] tested hypothesis: υ = 0; ne: not estimated.

Supplementary Table S3. Parameter estimates of the excess relative rate (ERR) models for leukemia (excluding chronic lymphocytic leukemia) and 2-year lagged cumulative red bone marrow dose in males only, SELTINE cohort, period 1968-2014.

**ERR model[a] (per Gy)**	**Coefficient**	**95%CI**	**AIC**	**p-value**
ERR(d) = βd	β =9.49	1.60; 21.36	1237.8	0.01[b]
ERR(d,a) = βd·a^{υ}	β = 3.31	0.94; 13.38	1235.4	<0.01[b]
	υ = 8.96	0.89; 17.57		
Restricted range of cumulative dose 0-100 mGy (n=149)	β = 1.37	0.06; 16.19	-	0.09[b]
First employment after 1956 (n=138)	β = 1.53	0.14; ne	-	0.04[b]
ERR(d,a,aex) = βd·a^{υ}·exp(μ·aex)	β = 3.22	0.92; 14.15	1237.4	-
	μ = 9.16	-0.81; 23.29		
	e^{υ} = 0.95	0.90; 8.05		
ERR(d,a) = (βd +γd^2)·a^{υ}	β = 4.13	2.63; ne	1236.9	0.45[c]
	γ = -11.06	ne; 48.05		
ERR(d,a) = βd·a^{υ} adjusted on neutron flag	β = 2.32	ne; 11.29	1236.2	0.20[d]
ERR(d,a) = βd·a^{υ} adjusted on internal contamination flag	β = 3.61	1.07; 14.21	1237.9	0.46[d]

[a] defined as λ_0(y,a,c,$duem$)[1 + ERR(d,cov)], where y is calendar year, a is attained age, c is company, $duem$ is duration of employment, d is cumulative colon dose, and cov is a set of covariables; aex: age at exposure; CI: likelihood-based confidence interval; AIC: Akaike information criteria; p-value of a likelihood ratio test vs. [b] tested hypothesis: β=0, [c] tested hypothesis: γ=0, [d] tested hypothesis: υ = 0; ne: not estimated.
